# C-Reactive Protein/Albumin Ratio Correlates With Disease Severity and Predicts Outcome in Patients With Aneurysmal Subarachnoid Hemorrhage

**DOI:** 10.3389/fneur.2019.01186

**Published:** 2019-11-12

**Authors:** Dingding Zhang, Huiying Yan, Yongxiang Wei, Xiangyu Liu, Zong Zhuang, Wei Dai, Jinsong Li, Wei Li, Chunhua Hang

**Affiliations:** Department of Neurosurgery, Nanjing Drum Tower Hospital, The Affiliated Hospital of Nanjing University Medical School, Nanjing, China

**Keywords:** aneurysmal subarachnoid hemorrhage, C-reactive protein, albumin, disease severity, outcome

## Abstract

**Aim:** The purpose of the present study was to determine if C-reactive protein (CRP)/albumin ratio was associated with disease severity and unfavorable outcome in patients with aneurysmal subarachnoid hemorrhage (aSAH).

**Methods:** One hundred and twenty-three consecutive patients suffering from aSAH were included in the study, which was carried out during the period of June 2016 to September 2018. Clinical and demographic parameters were recorded. CRP and albumin assessments were conducted upon admission. The association of CRP/albumin ratio with the disease severity and 3-month outcomes was evaluated.

**Results:** Higher CRP/albumin ratio was significantly associated with a higher World Federation of Neurological Surgeons Scale (WFNS) grade (*p* < 0.05). Poor outcome at 3 months was associated with a higher WFNS grade, higher serum glucose, higher CRP level, lower albumin level, higher Fisher score, higher CRP/albumin ratio, symptomatic cerebral vasospasm, intraventricular hemorrhage, delayed cerebral ischemia, and age using univariate analysis. The multivariate binary regression analysis revealed that the CRP/albumin ratio was independently associated with unfavorable outcomes after adjustment for age, WFNS grade, serum glucose, albumin, Fisher score, symptomatic cerebral vasospasm, intraventricular hemorrhage, and delayed cerebral ischemia.

**Conclusion:** Elevated CRP/albumin ratio was associated with disease severity and poor outcomes after aSAH.

## Introduction

Aneurysmal subarachnoid hemorrhage (aSAH) is a devastating form of stroke associated with high morbidity and mortality rates (1). Although the World Federation of Neurological Surgeons Scale (WFNS) and the Hunt and Hess Scale are the most commonly used SAH grading systems and serve as important prognostic factors for outcomes, neither system has achieved universal acceptance (2, 3). As a consequence, much effort has been directed toward the identification of blood-based biomarkers that are useful in predicting outcomes in aSAH patients (4). Among them, C-reactive protein (CRP) and albumin appeared to be the most promising biomarkers (5). High CRP levels have been studied in relation to prognosis and mortality in aSAH patients (6–8). Low serum albumin is known to be associated with poor prognosis and mortality (9). Recently, the CRP/albumin ratio has been established as an independent prognostic marker in patients with infection, malignancy, and critical illness (10–12). However, the prognostic value of the combined usefulness of CRP and albumin in aSAH has not been evaluated. Therefore, the aim of this study was to evaluate the correlation of CRP/albumin ratio with severity of disease and outcome in aSAH patients.

## Materials and Methods

### Patients

This study was a retrospective single-center study of all consecutive aSAH patients admitted to the Department of Neurosurgery in Nanjing Drum Tower Hospital, The Affiliated Hospital of Nanjing University Medical School between June 2016 and September 2018. The study was approved by the hospital's institutional review board. Our local institutional review board waived the requirement for informed consent since no intervention was performed and no personally identifiable information appeared. SAH was diagnosed by computed tomography (CT), and the diagnosis of aneurysm was achieved by digital subtraction angiography. Inclusion criteria were patients age 18 years and older, presentation to the hospital within 24 h after the onset of aSAH, and serum CRP and albumin levels measured upon admission. Exclusion criteria included SAH associated with autoimmune disease, inflammatory disease, liver disease, kidney damage, malnutrition, trauma, arteriovenous malformation, moyamoya disease, and SAH without identified source of bleeding using cerebral angiography. The selection of appropriate treatment modality (clipping or coiling) was consistent with latest guidelines (13, 14).

### Clinical Data

Collected data included patient characteristics on admission and during treatment course, radiological features, and clinical outcomes. SAH and the radiological severity were evaluated using the WFNS grading scale (15) and Fisher score, respectively. The incidence of cerebral vasospasm, acute hydrocephalus, and delayed cerebral ischemia (DCI) were also obtained for each patient. Cerebral vasospasm was defined according to previous studies (16, 17), and DCI was diagnosed according to previously published guidelines (18). Clinical outcomes were followed until death or 3 months after aSAH using the Glasgow Outcome Scale (GOS), with GOS 1–3 defined as poor and GOS 4–5 as good.

### Statistical Analysis

Continuous variables were expressed as a mean with standard deviation (SD) and were compared using the two-tailed Student's *t* test or Mann–Whitney *U* test, depending on data normality. Categorical variables are reported as count, and comparisons between groups were carried out using the chi-squared or Fisher test. Spearman rank correlation test was used to test the correlation of serum levels of the CRP/albumin ratio with the WFNS score. Analysis of the receiver operating characteristic (ROC) curves and the area under the ROC curves (AUC) were performed to evaluate the CRP/albumin ratio as predictive values for poor outcomes. In addition, we compared the ROC curves among serum CRP, glucose, albumin, and CRP/albumin ratio. A multivariate binary logistic regression analysis was performed on factors considered to be important to the univariate analysis. In this model, collinearity was examined with the variance inflation factor. The variable we used for multivariate analysis was variance inflation factor <5. Data were analyzed using the SPSS 21.0 statistical package (SPSS Inc., Chicago, IL), except the comparison of ROC curves, which was carried out using MedCalc statistical software version 18.9 (MedCalc Software, Mariakerke, Belgium). For all tests, *p* < 0.05 was considered as statistically significant.

## Results

A total of 152 patients suffering from aSAH over the period between June 2016 and September 2018 met the inclusion criteria. Among them, 29 patients were excluded from the study based on the above exclusion criteria, and 123 patients were finally included in the analysis. Patient characteristics, including age, sex, past medical history (hypertension and diabetes mellitus), clinical admission status, radiological features, development of cerebral vasospasm, DCI, CRP, and albumin levels, presence of acute hydrocephalus, and clinical outcome at 3 months post-SAH are presented in Table 1.

**Table 1:** Patient characteristics.

**Variable**	**Value**
No. of patients	123
Males/females	50/73
Mean age in years(range)	57.6 ± 11.1 (35–85)
Diabetes mellitus	4
Hypertension	49
WFNS grade	
1	23
2	31
3	28
4	19
5	22
Fisher score	
2	57
3	37
4	29
Aneurysm location	
Anterior	75
Posterior	35
Multiple aneurysms	13
GOS	
1–3	26
4–5	97
Repair procedure	
Clipping	9
Coiling	113
None	1
Acute hydrocephalus	5
DCI	23
Symptomatic cerebral vasospasm	18
Intraventricular hemorrhage	20
Glucose (mmol/L, mean ± SD)	6.628 ± 1.982 (3.07–13.72)
CRP, mean ± SD	15.829 ± 23.916(1.2–142.2)
Albumin, mean ± SD	40.350 ± 3.780(25.7–48.0)
CRP/Albumin ratio (× 1,000, mean ± SD)	0.419 ± 0.681(0.03–4.5)

Comparisons of demographic and clinical severity data between good and poor clinical outcomes in patients with aSAH are shown in Table 2. The glucose, CRP, and CRP/albumin ratio values on admission in patients with poor outcomes at 3 months were significantly higher than the corresponding values in patients with good outcomes (Table 2, *p* < 0.05). By contrast, patients who achieved good outcomes presented higher albumin level compared to those with poor outcomes (Table 2, *p* < 0.05).

**Table 2:** Univariate analysis comparing patients with poor and good 3-month outcomes.

**Variable**	**GOS (1–3)**	**GOS (4–5)**	**OR (95%CI)**	***P*-value**
Males/females	11/15	39/58	–	0.846
Mean age in years	60.923 ± 7.144	56.680 ± 11.789	1.036 (0.995–1.079)	**0.024**
Hypertension	12	37	–	0.459
Diabetes mellitus	0	4	–	0.578
WFNS			3.69 (2.205–6.173)	**<0.001**
1	2	21		
2	0	31		
3	3	25		
4	4	15		
5	17	5		
Fisher Score			2.263 (1.3–3.94)	**0.002**
2	8	49		
3	5	32		
4	13	16		
Aneurysm location			–	0.823
Anterior	17	58		
Posterior	7	28		
Multiple aneurysms	2	11		
Repair procedure (WFNS4–5)			–	0.095
Clip	5	1		
Coil	14	20		
None	1	0		
Acute hydrocephalus	3	2	–	0.107
DCI	12	11	6.701 (2.48–18.11)	**<0.001**
Symptomatic cerebral vasospasm	9	9	5.176 (1.794–14.939)	**0.003**
Intraventricular hemorrhage	8	12	3.148 (1.125–8.810)	**0.024**
Glucose (mmol/L, mean ±SD)	7.854 ± 2.272	6.299 ± 1.770	1.436 (1.153–1.789)	**<0.001**
CRP (mmol/L, mean ±SD)	43.881 ± 35.548	8.309 ± 11.295	1.078 (1.045–1.111)	**<0.001**
Albumin (mmol/L, mean ±SD)	37.696 ± 4.851	41.062 ± 3.101	0.79 (0.696–0.898)	**0.002**
CRP/albumin ratio (× 1,000, mean ±SD)	1.208 ± 1.047	0.207 ± 0.300	17.072 (5.159–56.491)	**<0.001**

*WFNS, World Federation of Neurological Surgeons; GOS, Glasgow Outcome Scale; DCI, Delayed cerebral ischemia. Bold values indicate p < 0.05*.

**Figure 1:**
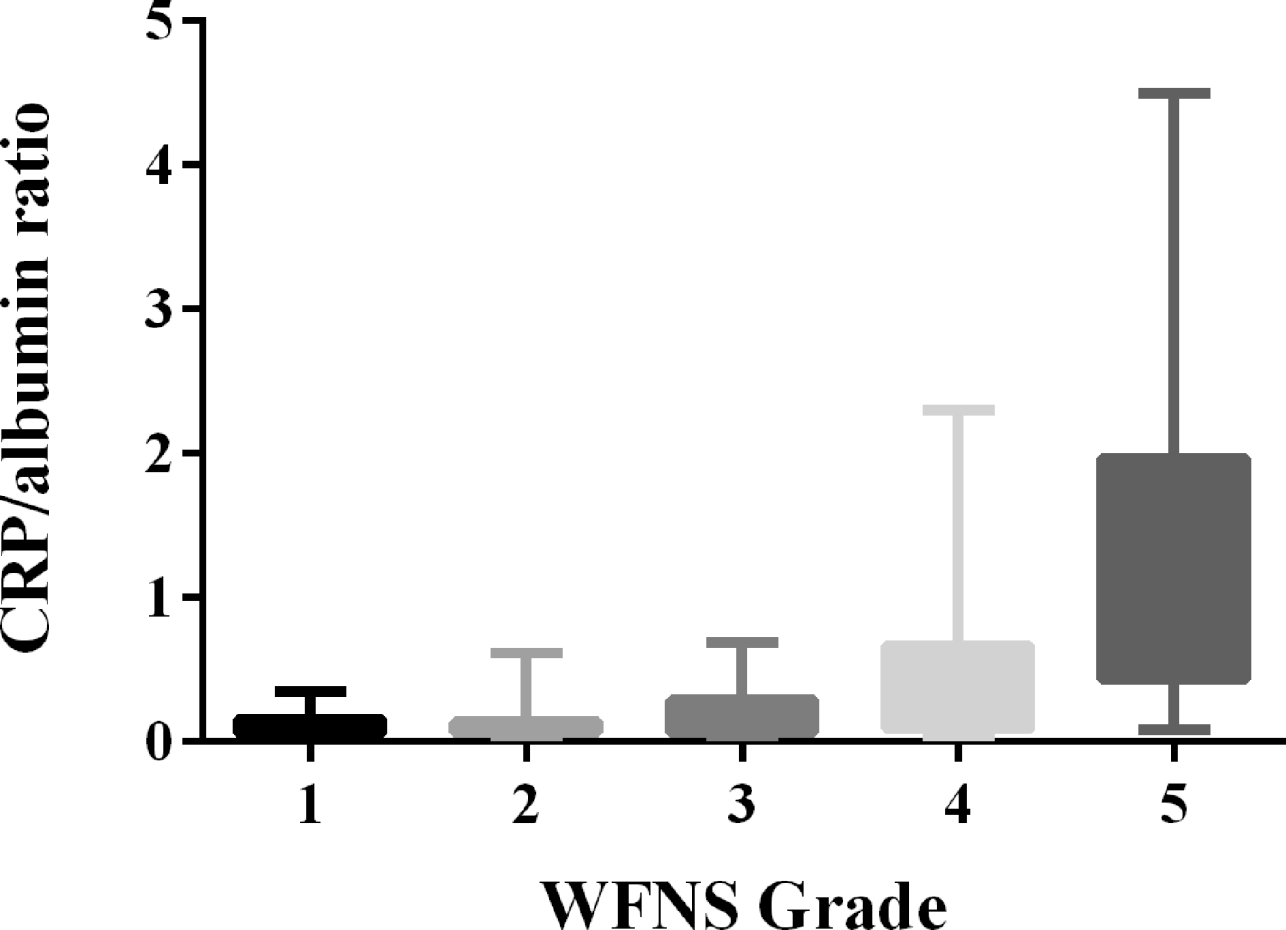
Correlation of the serum C-reactive protein (CRP)/albumin ratio with World Federation of Neurological Surgeons Scale (WFNS) grade after aneurysmal subarachnoid hemorrhage (aSAH) (*r* = 0.556, *p* < 0.001).

**Figure 2:**
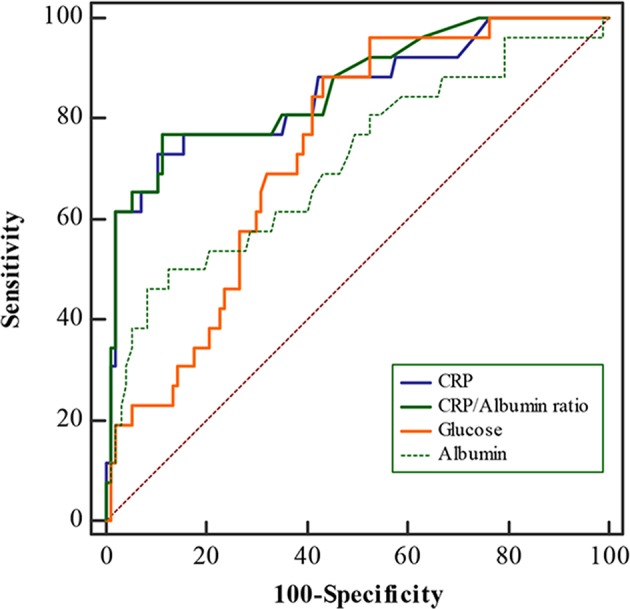
Receiver operating characteristic (ROC) curves for C-reactive protein (CRP)/albumin, CRP, albumin, and glucose levels for 3-month poor outcomes in aneurysmal subarachnoid hemorrhage (aSAH) patients. The area under the curve was calculated to be 0.862 for CRP/albumin, 0.851 for CRP, 0.712 for albumin, and 0.738 for glucose.

Admission CRP/albumin ratio levels were significantly correlated with WFNS grade (Figure 1, *p* < 0.05). ROC curves were compared to determine whether there was an additional benefit of using CRP/albumin ratio for predicting 3-month poor outcome. As shown in Figure 2, the area under the ROC curve of CRP/albumin ratio was higher than that of glucose (0.862 vs. 0.738, *p* = 0.047) and albumin (0.862 vs. 0.712, *p* = 0.028) for poor outcome prediction. These data indicate that the prognostic value of CRP/albumin ratio is more accurate than individual albumin and glucose. However, the ROC curve suggested that CRP/albumin ratio (0.862) had a similar predictive value to CRP (0.851) (*Z* = 1.107, *p* = 0.268).

Multivariate logistic regression analyses were used to reveal the independent predictors that showed an association in the univariate analyses (Table 2, *p* < 0.05). After adjustment for WFNS grade, serum glucose, albumin, Fisher score, age, symptomatic cerebral vasospasm, intraventricular hemorrhage, and DCI, the binary logistic regression identified CRP/albumin ratio to be independently related to poor outcomes in patients with aSAH (as shown in Table 3).

**Table 3:** Multiple binomial logistic regression analysis to predict poor 3-month outcome.

**Variables**	**Adjusted OR(95% CI)**	***P*-value**
Age	1.034 (0.967–1.106)	0.326
WFNS	1.840 (0.955–3.543)	0.068
Fisher score	0.881 (0.325–2.387)	0.803
DCI	2.200 (0.475–10.190)	0.314
Symptomatic cerebral vasospasm	1.564 (0.324–7.548)	0.578
Intraventricular hemorrhage	1.540 (0.307–7.716)	0.600
Glucose	1.207 (0.859–1.695)	0.279
Albumin	0.951 (0.802–1.126)	0.558
CRP/albumin ratio (× 1,000)	5.231 (1.113–24.585)	**0.036**

*CI, Confidence interval; OR, Odds ratio; WFNS, World Federation of Neurological Surgeons; DCI, Delayed cerebral ischemia; CRP, C-reactive protein. Bold values indicate p < 0.05*.

## Discussion

While CRP and albumin have been reported in association with the prognosis of aSAH, to the best of our knowledge, the present study is the first to examine an association of CRP/albumin ratio with the outcome of aSAH. The main results of this study indicated that serum CRP/albumin ratio was associated with the severity of aSAH. Moreover, elevated CRP/albumin ratio was significantly associated with unfavorable 3-month outcome, even after adjustment for several factors, including WFNS grade, serum glucose, albumin, Fisher score, age, symptomatic cerebral vasospasm, intraventricular hemorrhage, and DCI. In addition, the study demonstrated that the CRP/albumin ratio had stronger predictive potential for poor outcome in patients with aSAH, compared with glucose and albumin. These data indicated that the CRP/albumin ratio may be used as a prognostic tool in aSAH.

Previous studies have already demonstrated that hypoalbuminemia was not only common in aSAH patients but was also independently correlated with poor outcomes (9). Hypoalbuminemia in patients with aSAH may result from several mechanisms, including systemic inflammation, protein malnourishment, or deficiency in conjunction with hypercatabolic state (9). In our study, decreased albumin level was associated with poor outcomes, which was similar to that reported in previous trials (9). Although the exact mechanisms of hypoalbuminemia in aSAH patients remain elusive, a pilot study revealed that 1.25 g/kg/day albumin treatment was safe in SAH patients and might produce a better outcome (19). Importantly, some recent experimental evidence suggests that albuminemia mediates its neuroprotection through neurovascular remodeling and reducing cerebral lesions (20, 21). These findings may partly explain the neuroprotective effects of albumin in aSAH.

CRP is an acute-phase protein produced following stimulation by various cytokines in response to infection, ischemia, trauma, and other inflammatory conditions (22). High CRP levels have been shown to be associated with the severity of aSAH (5). Although the physiopathogenesis of the relationship between CRP and aSAH severity is not fully understood, many compelling studies have clearly indicated that CRP could also be a reliable prognostic factor for poor outcomes in patients with aSAH (6–8). Based on these observations, using the combination of CRP and albumin may provide more accurate information to help determine prognosis in aSAH than using either biomarker alone. In fact, the CRP/albumin ratio has been shown as an independent prognostic marker in patients with infection, malignancy, and other diseases (10–12). Thus, we assumed that increased CRP/albumin ratio may be superior to CRP and albumin alone in determining the severity and prognosis of aSAH. As described in former studies, we also observed a significantly increased CRP levels in the group of patients with poor outcomes. Moreover, we found that the CRP/albumin ratio was significantly increased in patients with severe WFNS grade, as well as in patients with poor outcomes. Furthermore, multivariate analysis revealed the CRP/albumin ratio as significant and independent predictor for unfavorable outcome in patients with aSAH. Finally, the predictive accuracy of CRP/albumin ratio was better than CRP and albumin level, as per the comparison of the ROC curves. These findings indicate that CRP/albumin ratio may serve as a novel and useful predictor for poor outcome in aSAH patients.

Elevated blood glucose is frequently detected early after aSAH, and glucose has been demonstrated to serve as a valuable prognostic factor for aSAH (23). In accordance with previous studies, our data also demonstrated that blood glucose was significantly increased in the poor outcomes group as compared with the good outcomes group. Meanwhile, we compared the prognostic value of CRP/albumin ratio and glucose in the present study. The ROC results showed that the AUC values of the CRP/albumin ratio were higher than glucose for predicting poor outcome after aSAH. After adjustment for WFNS grade, albumin, Fisher score, symptomatic cerebral vasospasm, age, intraventricular hemorrhage, and DCI, the CRP/albumin ratio was also an independent risk factor for poor outcome of aSAH. In contrast, glucose was not predictive for worse outcome after adjustment for the confounding factors. These data suggested that CRP/albumin ratio was a better indicator compared with glucose, which was revealed to be a significant risk factor for unfavorable outcome as well. The present study has several limitations. First, it was a retrospective study. Second, this single-center study lacks external validation. Therefore, the current results need further multi-institutional validation with larger samples. Another limitation of the study is the lack of prerupture measurements in the majority of patients. However, aSAH patients with autoimmune disease, kidney damage, inflammatory disease, and liver disease had been excluded from the present study. Finally, the follow-up period was 3 months, which is a relatively short-term follow-up study. Thus, in the future, it is necessary to perform long-term follow-up studies.

In summary, we found that CRP/albumin ratio significantly correlated with WFNS grade. In addition, the CRP/albumin ratio was strongly associated with poor prognosis of aSAH. Our results indicate that the CRP/albumin ratio could serve as a new, non-invasive, simple, economical, and feasible biomarker in predicting the severity and poor outcome of aSAH patients.

## Data Availability Statement

The datasets generated for this study are available on request to the corresponding author.

## Ethics Statement

The studies involving human participants were reviewed and approved by the institutional review board of Nanjing Drum Tower Hospital, The Affiliated Hospital of Nanjing University Medical School. Written informed consent for participation was not required for this study in accordance with the national legislation and the institutional requirements.

## Author Contributions

DZ designed the study, interpreted results, and wrote the manuscript. HY and WL collected the study data and interpreted results. YW, XL, ZZ, WD, and JL performed data analysis and revised the manuscript. CH designed and revised the manuscript.

### Conflict of Interest

The authors declare that the research was conducted in the absence of any commercial or financial relationships that could be construed as a potential conflict of interest.
